# *ACO*ustic: A Nature-Inspired Exploration Indicator for Ant Colony Optimization

**DOI:** 10.1155/2015/392345

**Published:** 2015-03-30

**Authors:** Rafid Sagban, Ku Ruhana Ku-Mahamud, Muhamad Shahbani Abu Bakar

**Affiliations:** ^1^Computer Science Department, University of Babylon, Babylon, Iraq; ^2^School of Computing, College of Arts and Sciences, Universiti Utara Malaysia, 06010 Sintok, Kedah, Malaysia

## Abstract

A statistical machine learning indicator, *ACO*ustic, is proposed to evaluate the exploration behavior in the iterations of ant colony optimization algorithms. This idea is inspired by the behavior of some parasites in their mimicry to the queens' acoustics of their ant hosts. The parasites' reaction results from their ability to indicate the state of penetration. The proposed indicator solves the problem of robustness that results from the difference of magnitudes in the distance's matrix, especially when combinatorial optimization problems with rugged fitness landscape are applied. The performance of the proposed indicator is evaluated against the existing indicators in six variants of ant colony optimization algorithms. Instances for travelling salesman problem and quadratic assignment problem are used in the experimental evaluation. The analytical results showed that the proposed indicator is more informative and more robust.

## 1. Introduction

Since the day of modelling “swarm intelligence” (SI) [1] as a computational technique for global optimization, ant colony optimization (ACO) algorithms went beyond being specific purpose methods. The first ACO algorithm, namely, ant system, was introduced as a novel SI metaheuristic for solving hard combinatorial optimization (CO) problems [2]. The idea is to consider the artificial ant processes that build solutions with the help of a common memory. The memory is readable and modifiable by all artificial ants. In ACO modelling, translating this natural optimization process into a combinatorial optimization system without monitoring causes one of the following situations. The first situation is that all ants may follow the same path. This results in an overexploitation state where the local optimum is occurring. The second situation is that the ants just perform a random walk. This results in an overexploration state [3]. The exploration and exploitation balance is needed for effective search not in ACO level, but at SI metaheuristic level.

In metaheuristics, higher level strategies of exploration are the typical solutions to escape from local optima and performing a robust search of a solution space [4]. Although the relevance of this concept is commonly agreed, so far, there is a limited unifying description to be found in the literature, while most of the work that deals with this topic focused on specific procedures [5]. Recently, reactive search [6] has emerged as a generic technique to improve the internal behavior of metaheuristics. It relies on exploration indicators to promote the exploration needed. In SI algorithms, prominent indicators are typically used for learning parameters [7, 8], determining when to restart the search [9, 10], or analyzing the explorative behavior of the algorithm [11, 12].

So far, utilizing traditional indicators does not satisfy the requirements of reactive search because they are simply not machine learning methods, except the indicator developed by Pellegrini et al. [13]. It utilizes an agglomerative clustering to quantify the exploration as the number of clusters of solutions visited. Two closest clusters can be concluded when the Euclidean distance between them is greater than a predefine threshold *ϵ*_*x*_ where *x*% of their solution components do not exist in the cluster. However, the definition needs to be reconsidered in terms of robustness against various circumstances because of the coherence with the magnitude of the distance matrix. For example, in ACO, the threshold *ϵ*_*x*_ is equal to 7.8 without local search, equal to 17.5 in with 2-opt local search, and equal to 35.8 with 3-opt local search. Therefore, this situation leads to an unstable measurement, especially when more rugged CO problem instances, such as QAP, need to be solved by SI algorithm.

This paper introduces, systematically, a step forward in proposing a statistical machine learning technique for indicating the exploration amount in ACO algorithm during the run. The idea of this work is simulated by a natural strategy, namely,* acoustical mimicry*, in which the signals exchanged between nest-mates in the nest may be mimicked by other insects that coexist with ants or interact with them as social parasites. In some way, the larva of the parasite is carried into the ant nest, where the ants treat it as a nest-mate. This biological strategy is utilized by the parasites to survive inside their host ant nests [14]. In this paper, the exploration conducted by artificial ants in the optimization process is the analogy of leaving the nest before parasites are killed by the real ants, that is, characterizing the current penetration state. The mechanism in nature is modelled into several statistical machine learning algorithms to detect the performance of the ACO algorithm.

The rest of the paper is organized as follows. The exploration measurement in ACO is overviewed in Section 2. The biological idea, the modelling, and the implementation of the proposed exploration indicator are illustrated in Section 3. The experimental setting is described in Section 4. The computational results are presented in Section 5. Finally, the conclusions and the suggested directions for future research are highlighted in Section 6.

## 2. Exploration Indicators in ACO

This section is to overview the existing techniques to indicate the exploration amount in ACO algorithm. Towards a reactive-based ACO search, by tuning an ACO algorithm, setting the parameters to values which allow an efficient search, describing the amount of exploration that an ACO algorithm performs, and detecting stagnation situations, the numerical exploration indicators are proposed [3, 6, 15]. The distance between solutions is one of the simplest indicators that calculate the number of arcs that are contained in one solution, but not in the other. It focuses on the duplicated arcs between two solutions, and if the degree of similarity is high, then the exploration amount is low [15]. Its disadvantage is being computationally expensive. Pellegrini [16] introduced the use of entropy indicator for characterizing the amount of exploration in ACO algorithm. Colas and Monmarch [17] employed entropy as a feedback indicator in their parameters control method proposal. This indicator can be applied simultaneously during the solution construction as follows. At each node, the ant calculates the selection probabilities of all other nodes in the following manner:

(1) $$\begin{array}{c} \varepsilon_{i}=-\overset{l}{\underset{j=1}{\sum }}p_{ij}\log p_{ij}, \end{array}$$

where *p*_*ij*_ is the probability of choosing arc (*i*, *j*) when being in node *i* and *l*, 1 ≤ *l* ≤ *n* − 1, is the number of possible choices. Therefore, finding the average of all entropies will be in the same way. Another indicator, convergence factor, is introduced by Blum [18] to be used in a hypercube framework [19]. It is used to track the convergence of ants, which is the converging phase of all ants. The convergence factor is calculated as follows:

(2) $$\begin{array}{c} \frac{\sum_{\tau_{ij}\epsilon T}\min\left\{\tau_{\max}-\tau_{ij},\tau_{ij}-\tau_{\min}\right\}}{n^{2}}, \end{array}$$

where *T* is the pheromone matrix and *τ*_*ij*_ is the pheromone trails. Another tool for indication is acceptance criteria (AC), which is used with restart strategies to increase the exploration amount in ACO. It is defined by Stützle [20] as such: if the *i*_last_ was the iteration counter *i* in which the best-iteration solution has been found, then the optimal point for restarting the search can be calculated as follows:

(3) $$\begin{array}{c} \mathrm{AC}=\left\{\begin{array}{cc} s^{\prime \prime } & \text{if}\;\;f\left(s^{\prime \prime }\right)<f\left(s\right)\\ s^{\prime \prime \prime } & \text{if}\;\;f\left(s^{\prime \prime }\right)\geq f\left(s\right),\;\;i-i_{\text{last}}>i_{r}\\ s & \text{otherwise}. \end{array}\right. \end{array}$$

Based on this formula, the value of *s*′′′ is generated randomly by a new initial solution which corresponds to a restart of the algorithm. The amount of exploration will increase without guaranteeing that the same regions will not be visited again. The problem has been solved by Sagban et al. [10]. Solnon and Fenet [12] used the similarity ratio to indicate how much the solutions are similar. This is done by finding the final solutions generated by ants and then applying some statistics tools such as the standard deviation and the variation coefficient. One example of these statistics is the standard deviation of the objective function of solutions constructed. The exploration amount will be high if the standard deviation is near to one and low if near to zero. Gambardella and Dorigo [21] introduced the average lambda branching factor (*λ*-branching) which depends directly on the pheromone trail values and makes it more suitable for tracking the ant's behavior while the computation is going on. This technique measures the diversity of the pheromone trail values in a more direct way. It does not change much from iteration to iteration. The branching factor can be defined as follows. Let *ph*_max⁡(*i*, *j*) and *ph*_min⁡(*i*, *j*) be the maximum and minimum pheromone amount, respectively, of all the arcs that exit from node *i*. Let *d* be the difference between two amounts. The branching factor of node *i* is the number of arcs that is greater than *λ*∗*d* + *ph*_min⁡(*i*, *j*), where 0 ≤ *λ* ≤ 1. The average of the lambda branching factor of all nodes gives an indication of the amount of exploration conducted by each ant. The *λ*-branching indicator is very important in the experimental history of improving the ACO metaheuristic. Recently, Neyoy et al. [22] and Neyoy et al. [7] used this indicator in designing fuzzy controllers for parameters adaptation in ACO algorithms. The disadvantage of the branching factor is its dependency on the value of parameter *λ*. Moreover, it is not applied for other CO problems such as quadratic assignment problem. Pellegrini and Favaretto [5] quantified the exploration as the number of clusters of solutions visited: *E*(*B*, *I*, *R*, *h*) = |*L*(*B*, *I*, *R*, *h*)|, where *L* is the set of clusters resulting from the solutions visited by the algorithm *B* when solving the instance *I* using the resources *R* and the seed *h*. Given such definition, it is possible to observe the behavior of any metaheuristic without needing a deep knowledge on the internal state of the algorithm.

## 3. The Proposed Indicator:* ACO*ustic

The nature-inspired indicator, denoted as (*ACO*ustic), is proposed to characterize the exploration in the ACO algorithm. To present the idea, the following subsections are discussed: the biological schema, the modelling, and the implementation.

### 3.1. The Biological Schema

**Figure 1:**
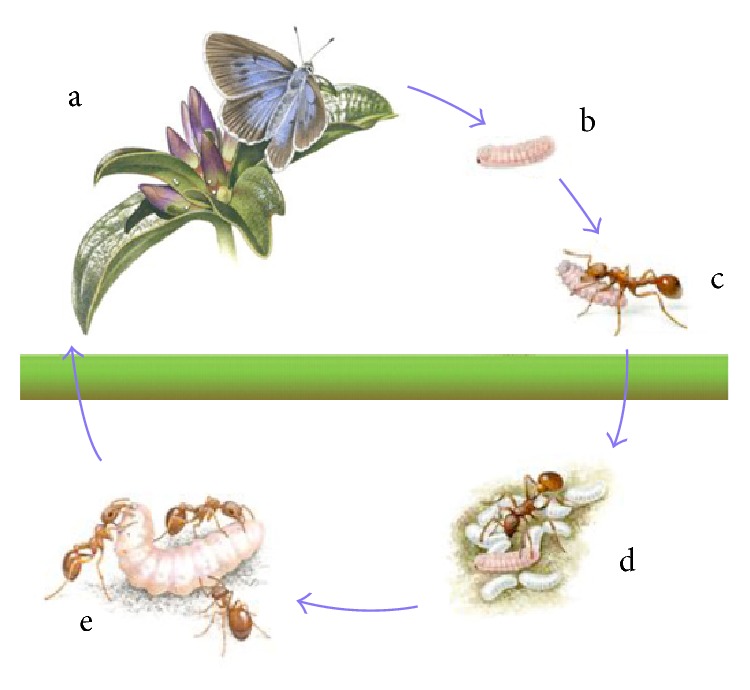
The* Myrmica* ants-*Maculinea* larvae system.

Rapid and effective communication between ants is a key attribute that enables them to live in dominant, fiercely protected societies.* Myrmica* ant colonies, in particular, are exploited by social parasites called* Maculinea* butterflies [23]. The process of* Trophallaxis* (i.e., distributing liquid food from the “social stomach”) between attendance worker and other nest-mates is the main process in food foraging behavior of ants. The worker ants produce acoustics during the process. The* Maculinea* larvae are interfering with the* Myrmica* system and produce similar acoustics to that of the colony. The high number of worker ants leads to a low relatedness between nest-mates. A greater variance in nest-mates acoustic signals leads to a higher likelihood of being infested [14]. Through this indicator, the larva can decide the optimal point to leave the colony before it is discovered by other ants as depicted in Figure 1.

**Figure 2:**
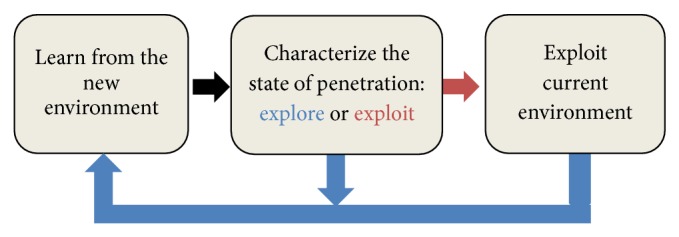
The scheme of acoustical indication in nature.

The larva is able to evaluate the situation inside the nest whether to leave or to stay. If the relatedness between nest-mates becomes high, then the likelihood of being clustered around the larvae will become low. This is an indication for the larva to explore another nest before being killed; otherwise the larva will continue to exploit the current nest until further notice. The acoustic reaction in this process can be simplified in three basic components as shown in Figure 2.

In ACO modelling, a colony of artificial ants inspires its characteristics from the real ant's foraging behavior. The construction graph simulates the environment that ants and larvae agents are moving on. For larvae agents, the interaction with the new environment is highly related to the state of penetration, that is, the learning process. The agents can decide whether to continue with the current exploitation or to explore another environment. To simulate the process of characterizing the state of penetration, statistical analyzing and agglomerative clustering algorithms are developed in this paper.

### 3.2. The Modelling

In this section, the way of characterizing the state of penetration is used as a didactic tool to explain the idea behind the* ACO*ustic's proposal. The behavior of ACO algorithm is described in terms of exploration and exploitation processes. According to the scheme described in Figure 2, the natural scheme in parasites-ants system translates into problem solving models as follows.

Let a construction graph *G* = (*N*, *A*) represent a CO problem, where *N* is the set of nodes; *A* is the set of arcs; |*A*| = *a* and |*N*| = *n*. The fitness landscape of the given CO problem is defined by the following: *P* is a population set which includes all solutions to the CO problem, where each solution *s* ∈ *P* assigns a fitness value *f*(*s*) and has a structure of neighborhood *N*⊆*P* × *P*. A colony of artificial ants performs a biased walk in this landscape with the goal of finding low *f*(*s*) (in case of minimization problems). The set *C*_*p*_(*t*) represents the collection of acoustics (sounds) that emanate from the landscape traversed by the ants of a perspective colony at time *t* where *C*_*p*_(*t*)⊆*P*(*t*) × *P*(*t*) where *c*_*i*_ and *c*_*i*+1_ are two acoustics belonging to *C*_*p*_(*t*) where *c*_*i*_ = {*x*_1_, *x*_2_,…, *x*_*a*_}; *c*_*i*+1_ = {*y*_1_, *y*_2_,…, *y*_*a*_} where the length of each acoustic signal is equal to *a*. The relatedness between two nest-mates is defined by the similarity between their acoustics. Two acoustics *c*_*i*+1_ and *c*_*i*_ are considered as being similar if their similarity neighborhood SN is below a predefined threshold *X*: 
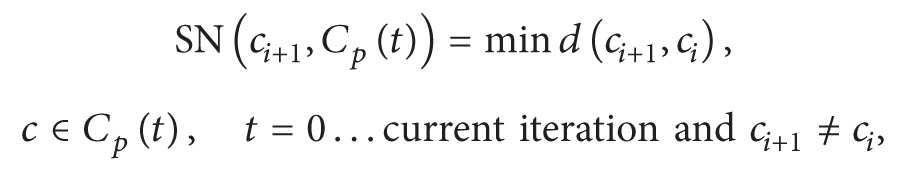
(4)

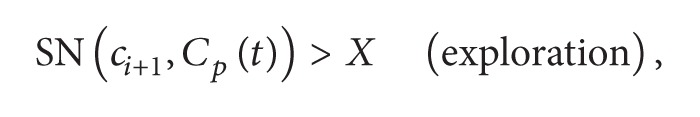
(5)

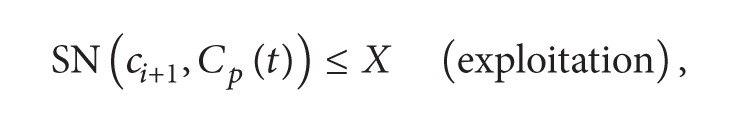
(6)where *d* is the Euclidian distance between two acoustics in *C*_*p*_(*t*) within Euclidian space *R* ^*n*^. The exploration occurs when SN of the two acoustics is greater than the boundary of neighbourhood threshold (*X*); otherwise it is identified as exploitation.

A population-based memory scheme is used to record the best-iteration solutions produced by the algorithm during the run. An agglomerative clustering procedure is applied to the recorded population every ten (10) iterations. This is to determine the similarity features of the population through its acoustics during the past ten iterations. A matrix of distances is defined to conduct the clustering and then detect the number of clusters. The Euclidean distance *d* between *c*_*i*_ and *c*_*i*+1_ is a common way for finding similarity as follows:

(7) $$\begin{array}{c} d\left(c_{i},c_{i+1}\right)=\sqrt{\overset{a}{\underset{j=1}{\sum }}{\left(x_{j}-y_{j}\right)}^{2}}. \end{array}$$

The quantity *d* may have different magnitudes so that it is normalized to the size of population:

(8) $$\begin{array}{c} d_{\mathrm{norm}}=\frac{d}{\left|P\right|}. \end{array}$$

Three statistics medians (mean, variance, and standard deviation) are derived in (9), (10), and (11), respectively:

(9) $$\begin{array}{c} \text{mr}\left(t\right)=\overset{\max-1}{\underset{i=1}{\sum }}\;\overset{\max}{\underset{j=i+1}{\sum }}\frac{d_{\mathrm{norm}}}{\left(\left(\max^{2}-\max\right)/2\right)}, \end{array}$$

(10) $$\begin{array}{c} \text{vr}\left(t\right)=\overset{\max-1}{\underset{i=1}{\sum }}\;\overset{\max}{\underset{j=i+1}{\sum }}{\left(C_{ij}-\text{mr}\right)}^{2}, \end{array}$$

(11) $$\begin{array}{c} \text{stdr}\left(t\right)=\sqrt{\frac{\text{vr}}{\left(\max-1\right)}}, \end{array}$$

where max is the maximum size of the distance matrix. In order to minimize the computational efforts and keep the algorithm nonweights, the size of matrix fixes to (10). The agglomerative hierarchical technique uses for calculating the number of clusters are as follows:

(12) $$\begin{array}{c} C_{\text{Num}}\left(C_{p}\left(t\right)\right)=\left|L\left(C_{p}\left(t\right)\right)\right|, \end{array}$$

where *L* is the set of clusters resulting from the solutions visited by the ants. The statistics and clustering information are combined. The relatedness between ants can be calculated by finding the difference between the mean of distances and the number of clusters by the standard deviation of distances as follows:

(13) $$\begin{array}{c} \text{rltdnss}=\frac{\left(\text{mr}-C_{\text{Num}}\right)}{\text{stdr}}. \end{array}$$

The definition of exploration and exploitation in (5) and (6) can be reformulated based on the relatedness between acoustics (rltdnss) as follows:

(14) $$\begin{array}{c} \text{rltdnss}>{NC}_{\text{throushold}}\;\left(\text{exploration}\right),\\ \text{rltdnss}\leq {NC}_{\text{throushold}}\;\left(\text{exploitation}\right), \end{array}$$

where *NC*_throushold_ is the lowest degree of clustering. It is detected by capturing the first value of rltdnss within the first ten iterations. For instance, when *C*_Num_ is decreased from 10 to 8, this indicates that the exploitation has occurred. In contrast, if *C*_Num_ stays as it is, this indicates that the exploration has occurred. The assignment of *NC*_throushold_ has to be complete within the first ten iterations. Hereafter, each new value of rltdnss will be characterized as either exploration or exploitation accordingly.

### 3.3. The Implementation

This section walks through the implementation of* ACO*ustic algorithm. The pseudocode of the algorithm is illustrated in Pseudocode 1.

The nearest neighborhood threshold *X* is entered, the vector of acoustics clusters *C*_*i*_ is defined, and other variables such as miniDist, *C*_Num_, *NC*_throushold_, and max are initialized as in Procedure 1.

In findSimilarities algorithm, a matrix of Euclidian distances between acoustics is generated and the statistical medians are calculated as in Pseudocode 2.

In determineRelatedness, the minimum distance miniDist is calculated from the distance matrix that is generated earlier. The nearest two clusters are united, the distance matrix is recalculated, and finally miniDist and the number of clusters *C*_Num_ are updated as in Pseudocode 3.

In Pseudocode 3, the number of clusters and the statistics collected earlier are combined and returned as a relatedness quantifier denoted as rltdnss.

## 4. Experimental Setting

In this section, the experimental design for applying the* ACO*ustic for several standard ACO algorithms is described. The aim of the application is to examine the ability of* ACO*ustic for the following: (i) monitoring its performance against different ACO algorithms, (ii) monitoring its performance against different fitness landscapes, and (iii) evaluating its performance against the state-of-the-art measurement tools in ACO. The implemented ACO algorithms are ant system (AS), elitist ant system (EAS), ant colony system (ACS), rank-based ant system (RAS), max-min ant system (MMAS), and best-worst ant system (BWAS). Two different fitness landscapes are used: travelling salesman problem (TSP) and quadratic assignment problem (QAP). The performance is reported to have been compared with average *λ*-branching and exploration quantifier as measurement tools. In the comparisons, the effect of the parameters of MMAS algorithm on the exploration and exploitation mechanisms is analyzed; several scenarios have been considered and the effect of the raggedness of fitness landscape is analyzed.

The analyzed parameters are pheromone intensity (*α*), preheuristic importance (*β*), evaporation rate (*ρ*), and the number of ants (*m*). The parameters setting suggested by Pellegrini et al. [24] has been considered. The stopping criteria are either the completion of 350 sec (only the first 3000 iterations are reported) for large instances or finding the optimal solution for small instances. The C coding is used in the implemented algorithms. The experiments are conducted on a Windows 8 64-bit operating system, processor Intel Core i3-3217U with CPU @ 1.80 GHz, RAM 4 GB. Each experiment is executed ten times to avoid the stochastic behavior. The main results of this application are depicted as below. The TSP instances used in the experiments are selected from TSPLIB repository and from the 8th DIMACS challenge. Following the TSPLIB format, d198 instance is selected. Following the DIMACS format, one random instance is generated using* portgen*; the instance generator is adopted in the 8th DIMACS TSP challenge. It is generated with size = 2000 and seed = 39200. The* kra30a.qap* instance used in the experiments is selected from QAPLIB repository.

## 5. Computational Results

**Figure 3:**
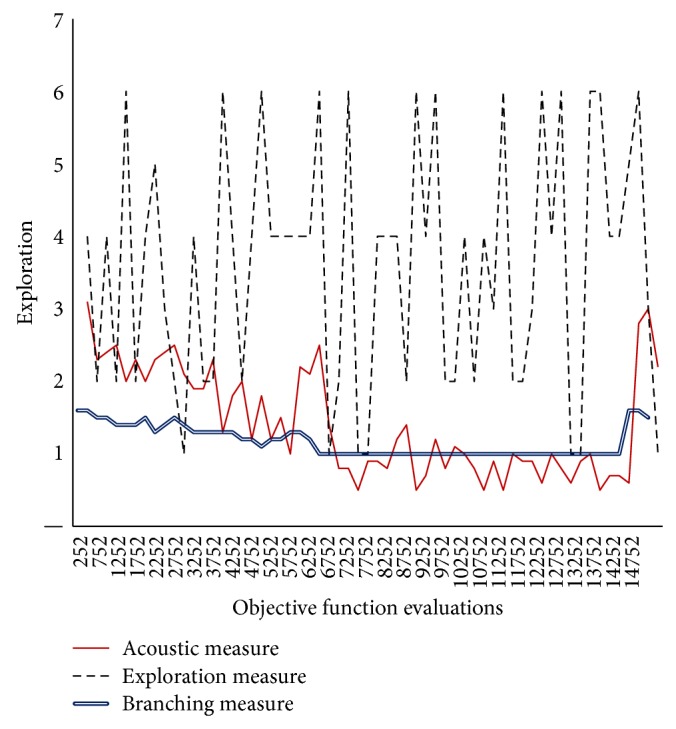
Evaluating* ACO*ustic against traditional indicators in ACO for the TSP instance d198 with nearest neighbourhood threshold = *ϵ*8.

**Figure 4 fig4:**
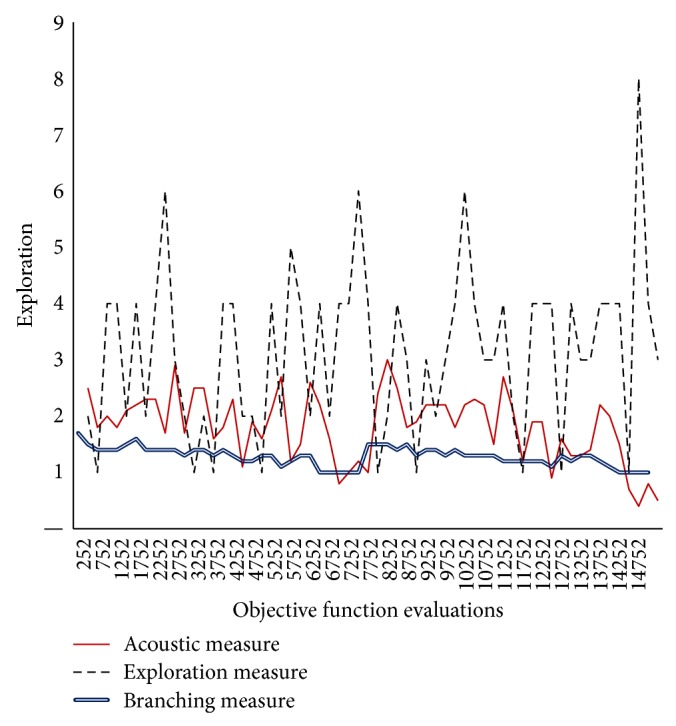
Evaluating* ACO*ustic against traditional indicators in ACO for the TSP instance d198 with nearest neighbourhood threshold = *ϵ*7.

**Figure 5:**
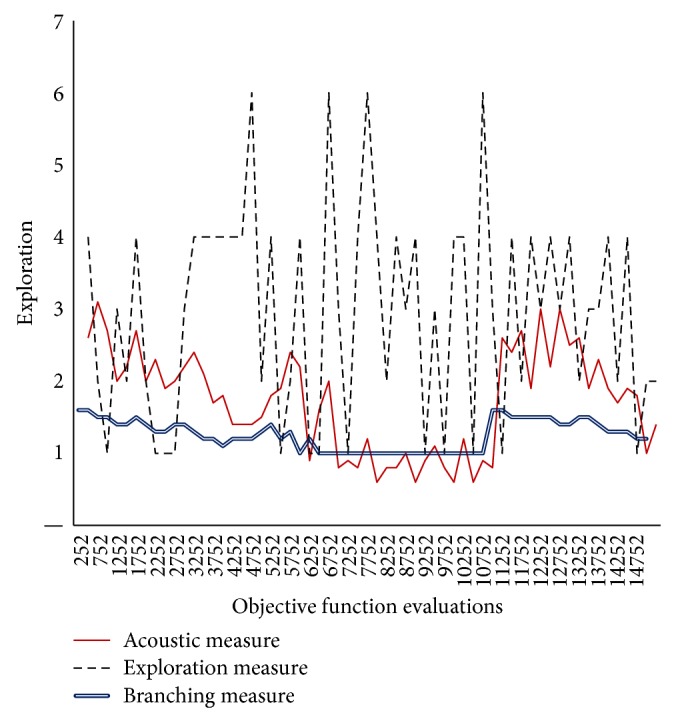
Evaluating* ACO*ustic against traditional indicators in ACO for the TSP instance d198 with nearest neighborhood threshold = *ϵ*6.

**Figure 6:**
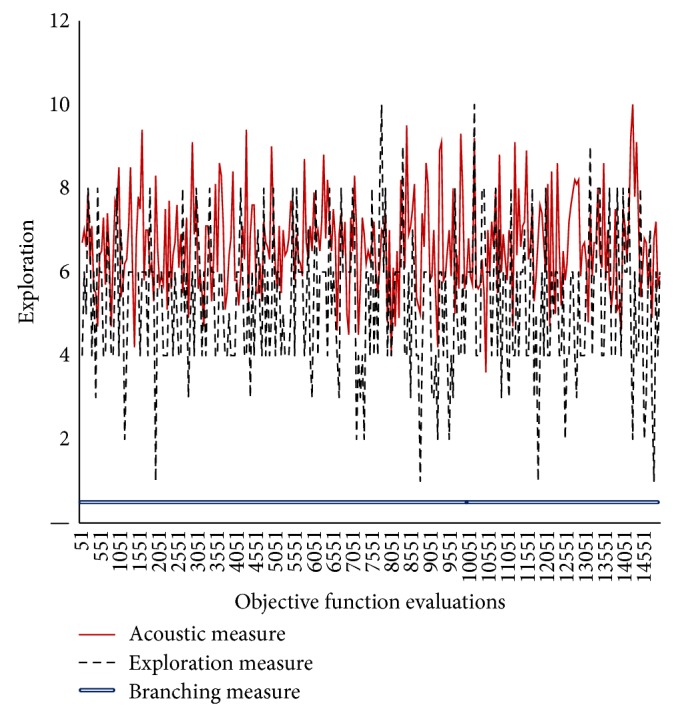
Evaluating* ACO*ustic against traditional indicators in ACO for the QAP instance kra30a with nearest neighborhood threshold = *ϵ*8.

**Figure 7:**
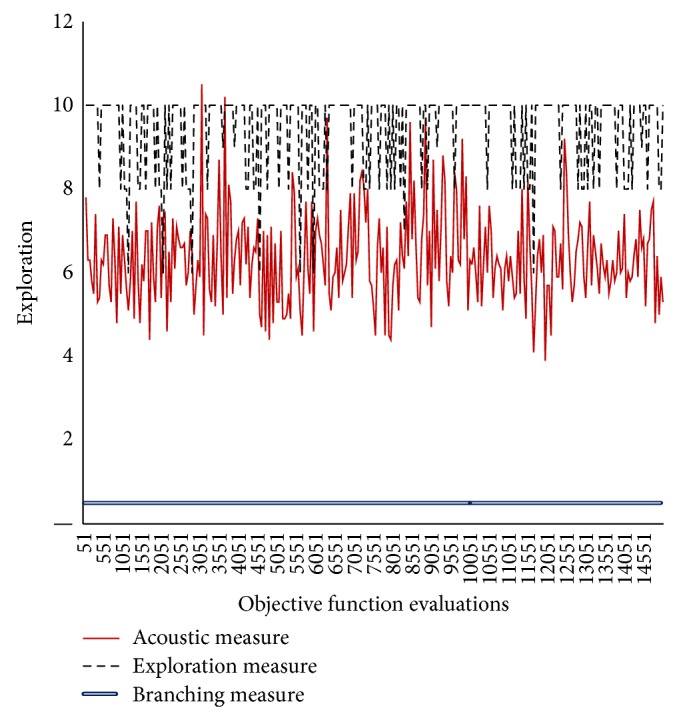
Evaluating* ACO*ustic against traditional indicators in ACO for the QAP instance kra30a with nearest neighborhood threshold = *ϵ*7.

**Figure 8:**
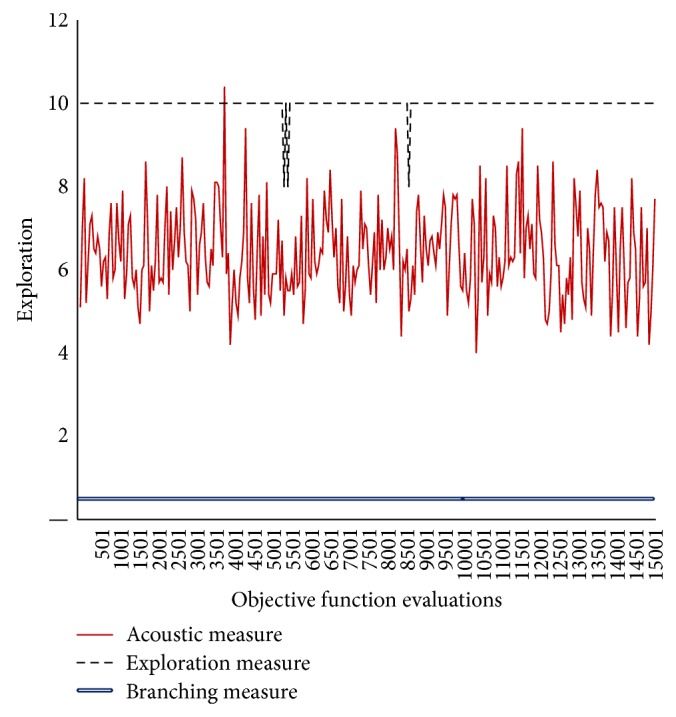
Evaluating* ACO*ustic against traditional indicators in ACO for the QAP instance kra30a with nearest neighborhood threshold = *ϵ*6.

**Figure 9:**
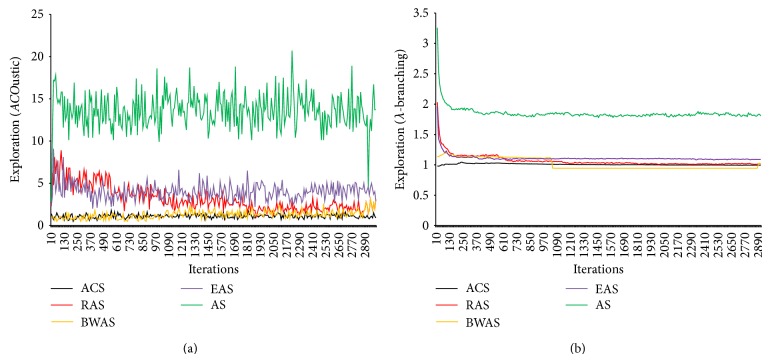
The performance of five ACO algorithms using the* ACO*ustic (a) and branching factor indicator (b) for a DIMACS instance size = 2000 and seed = 39200 generated using portgen.

**Figure 10:**
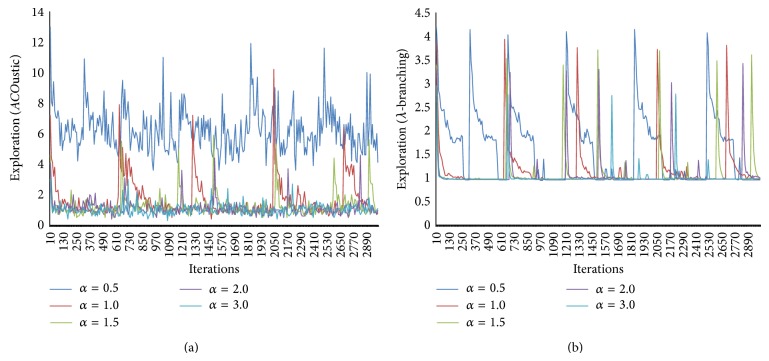
The effect of pheromone intensity (alpha) measured by* ACO*ustic (a) and measured by *λ*-branching (b).

**Figure 11:**
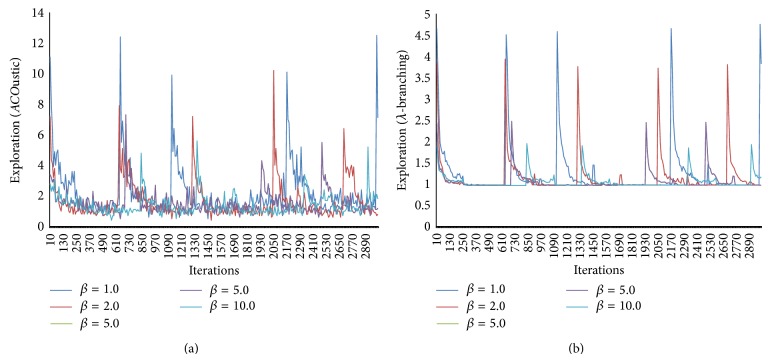
The effect of preheuristic (beta) measured by* ACO*ustic (a) and measured by *λ*-branching (b).

**Figure 12:**
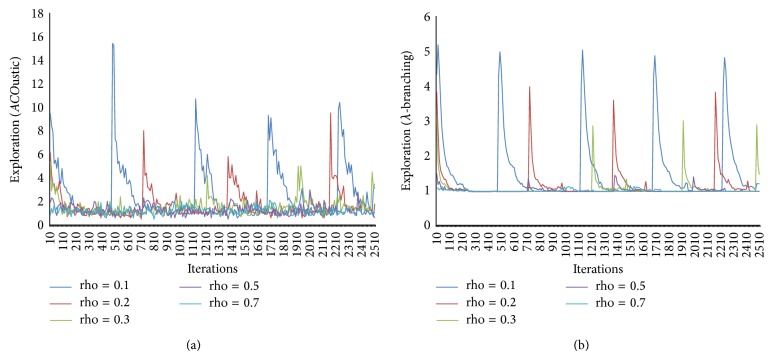
The effect of preheuristic (beta) measured by* ACO*ustic (a) and measured by *λ*-branching (b).

**Figure 13:**
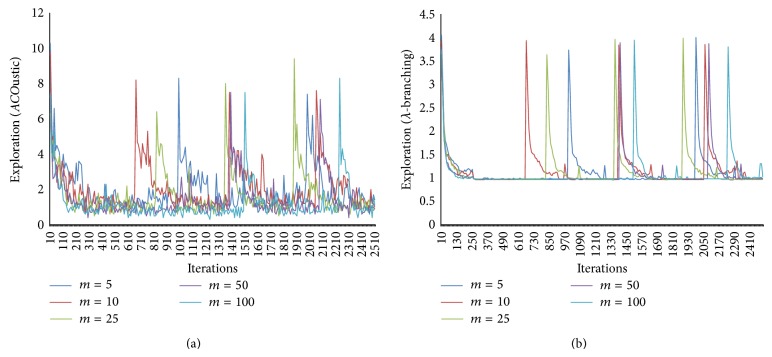
The effect of number of ants (*m*) measured by* ACO*ustic (a) and measured by *λ*-branching (b).

This section presents the results of two types of experiments that have been conducted to evaluate the performance of* ACO*ustic indicator. The first type of results reported the robustness of the proposed tool against the difference in the raggedness of TSP and QAP fitness landscapes (see Figures 3–8). The second type of results reported the ability of the proposed tool to analyze the convergence behavior under two conditions, the convergence of different ACO algorithms (see Figure 9) and the convergence of different parameters settings for MMAS, the prominent ACO algorithm (see Figures 10–13).

In Figures 3–8, the *y*-axis visualizes the explorative behavior of MMAS using the* ACO*ustic indicator compared with *λ*-branching indicator and exploration indicator. The *λ*-branching indicator is selected for comparison due to its theoretical and practical importance as a traditional indicator in ACO literature. The exploration indicator is selected because of its similarity to* ACO*ustic in the context of being a machine learning method. The *x*-axis represents the number of objective function evaluations. The general performance of the MMAS algorithm is reported for TSP and QAP.

Figures 3 [Fig fig4]–5 showed that when different values of neighborhood threshold are used, the proposed indicator gives the same insights into the shape of fitness landscape for TSP that other traditional indicators gave. In fact, traditional indicators have no problem of robustness when applied for TSP problem solving. Therefore, when 80%, 70%, or 60% of the components of one TSP solution do not exist in the other solution, the indication is similar. This confirms that* ACO*ustic retains the same quality of indication of other indicators.

Figures 6–8 showed that, for QAP, the* ACO*ustic indicator shows significant robustness against the branching indicator and exploration indicator. In Figure 6, when 80% of the components of one QAP solution do not exist in the other solution, the indication of* ACO*ustic and exploration ranges between zero (0) and eight (8). In Figure 7, when the rate is deduced to 70%, the disparity in the indication of* ACO*ustic and exploration begins where the range of the exploration amount in the latter indicator tends to be more towards ten (10). In Figure 8, when the rate is reduced to 60%, the sensitivity for exploration behaviour was at its lowest level. On the other hand, the branching factor in all three cases is the same, that is, equal to one (1), while the relatedness of* ACO*ustic ranges between four (4) and eight (8).

As shown, different values of neighborhood threshold give the same insights into the shape of fitness landscape for QAP that other traditional indicators did not give. In fact, the behavior of* ACO*ustic relies strongly on the normalized quantities of the distance between solutions and the number of clusters. These are statistical and clustering information about the current population. Hence, by normalization, the influence of the difference in magnitudes of the distance matrix has been reduced. This confirms the problem of robustness in traditional indicators when applied for QAP problem solving.

In Figures 9–13, the general performance of AS, EAS, RAS, ACS, and BWAS algorithms and the effect of parameters on MMAS behavior are reported. The *y*-axis visualizes the exploration using* ACO*ustic indicator and *λ*-branching indicator for TSP. The *x*-axis represents the number of iterations.

In Figure 9, the results of analyzing using the proposed indicator showed that AS tends to be a very explorative algorithm. The rest of the tested algorithms either start with a very short exploration phase followed by a very aggressive exploitation phase (e.g., EAS and RAS) or skip the initial exploration phase (e.g., ACS and BWAS). This is mainly achieved by a stronger emphasis given to the best tours found during the search. In general, when compared with *λ*-branching indicator, the proposed indicator draws the same shape for TSP.

In Figure 10, the effect of varying the main parameters on the explorative and exploitative behaviors of MMAS algorithm is characterized. The proposed indicator can detect the relationship between parameter values and local search.  Figure 10(a) showed clearly that the higher the value of pheromone intensity, the lower the exploration. The value *α* = 1.0 is the ideal value to achieve a moderate behavior.

In Figure 11, the influence of preheuristics parameter is tested. The higher the value of the parameter (*β*) is, the more greedy the behavior is recorded to become at its peak when (*β* = 10).

In Figure 12, the evaporation ratio as a trail learning factor has distinct impact on the way of search. Increasing the value of rho results in slow learning of pheromone trail parameters, and thereby the chance of forgetting the previous search experience will increase. In this way, the value of rho = 0.7 leads to the increase of exploitation while the value of rho = 0.5 seems ideal.

Figure 13 showed that the exploration decreases with respect to the number of ants. The greater the number of ants, the lower the number of iterations performed in a run and consequently the lower the number of different probability distributions used. A high value of *m* implies that the likely edges are often the same. In general, when compared with *λ*-branching indicator, the proposed indicator draws the same shape for TSP which reflects the explorative/exploitative behavior influenced by parameter tuning.

As shown, these insights are compatible with the common beliefs among ACO researchers. The* ACO*ustic indicator is a very convenient tool for characterizing the exploration on-demand. This conclusion came as a result of its robustness against the state-of-the-art indicators in ACO and of using the statistical machine learning paradigm.

## 6. Conclusion

Towards a reactive-based ACO search, the* ACO*ustic is proposed. It is a nature-inspired exploration indicator that helps in measuring the amount of exploration, detecting stagnation, tuning the algorithm, adapting parameters in an online manner, and analyzing the behavior of the algorithm. The biological idea that inspired the proposed indicator is presented, modelled, and implemented. The comparison with the state-of-the-art indicators in ACO showed that the* ACO*ustic is more informative and more robust.

For future work, the proposed exploration measurement will be harnessed in automating the exploration and exploitation in reactive-based ACO algorithms. The proposed tool can be easily applied for other metaheuristics and local search algorithms.

## Figures and Tables

**Pseudocode 1:**
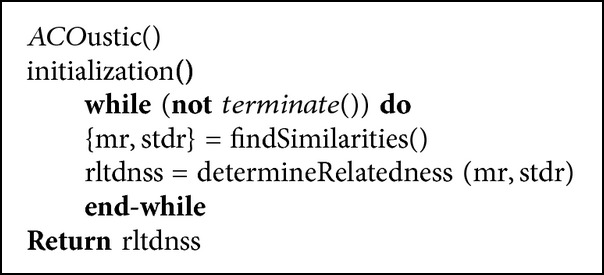
The pseudocode of *ACO*ustic algorithm.

**Procedure 1:**
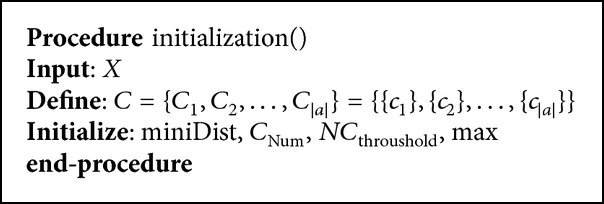
The initialization procedure.

**Pseudocode 2:**
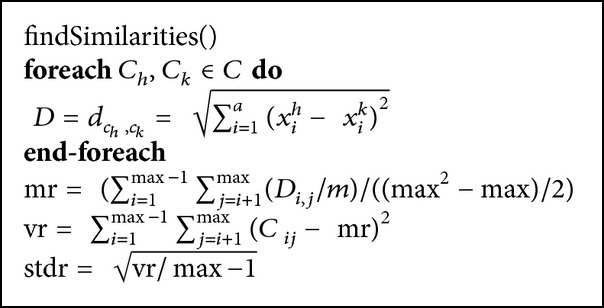
The pseudocode of findSimilarities algorithm.

**Pseudocode 3:**
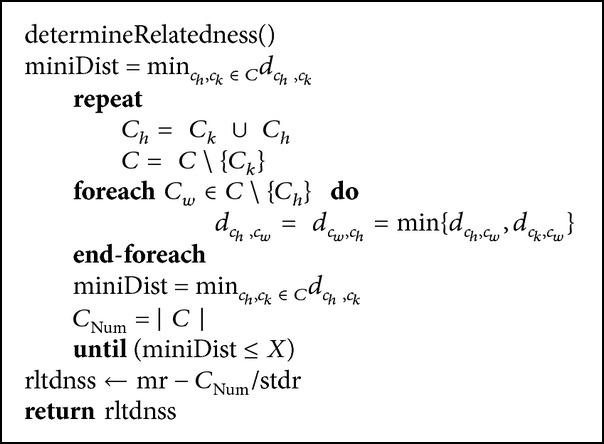
The pseudocode of determineRelatedness algorithm.
